# Supplementary material for: Environmental factors influencing the prevention of secondary health conditions among people with spinal cord injury, South Africa
Source: PLoS One. 2021 Jun 25;16(6):e0252280. doi: 10.1371/journal.pone.0252280 (PMC8232458; doi:10.1371/journal.pone.0252280)
Supplement: Attachment — Submitted filename: Response to Reviewers27042021.pdf [file pone.0252280.s002.pdf]

Physiotherapy

Faculty of Health Sciences · Private Bag 3, Wits, 2050, South
Tel: +27 11 717 3702 · Fax: 27 86 553 4762 · Email: sonti.pilusa@wits.ac.za

27 April 2021

Dear Reviewers

Re: Corrections of the manuscript PONE-D-20-20175R1

Thank you for the feedback received on my manuscript.

Please see Table 1 with point by point corrections,

Corrections - highlighted in yellow

**Comments**	**corrections**	**Line number**
**Editor**		
Please review your reference list to ensure that it is complete and correct. If you have cited papers that have been retracted, please include the rationale for doing so in the manuscript text, or remove these references and replace them with relevant current references. Any changes to the reference list should be mentioned in the rebuttal letter that accompanies your revised manuscript. If you need to cite a retracted	Corrected<br>Citation (29)- removed this reference and replaced with a more recent article<br>29. Maart S, Eide AH, Jelsma J, Loeb ME, Toni MK. Environmental barriers experienced by urban and rural disabled people in South Africa. Disabil Soc. 2007;22(4):357–69.<br>Replaced with:<br>Hanass-Hancock J, Nene S, Deghaye N, Pillay S. 'These are not luxuries, it is essential for access to life': Disability related out-of-pocket costs as a driver of economic vulnerability in South Africa. African J Disabil [Internet]. 2017 [cited 2017 Nov 1];6(a280):1–10. Available	Line 268

article, indicate the article’s retracted status in the References list and also include a citation and full reference for the retraction notice.	from: https://doi.org/10.4102/ajod.v6i0.280%0A<br><br>Citation (8)<br>8. Mashola MK, Mothabeng J, Olorunju S. Readmission dues to secondary health conditions in people with spinal cord injury at a private rehabilitation facility in South Africa.Poster at WCPT congress 2017, Cape Town. 2017;(21):2017.<br>This reference is for a conference poster. Replaced the article with the article by Mashola et al. 2019 (the same information from the same author)<br>**Replaced with:**<br>Mashola MK, Olorunju SAS, Mothabeng J. Factors related to hospital readmissions in people with spinal cord injury in South Africa. South African Med J [Internet]. 2019 Jan 31;109(2):107. Available from: https://doi.org/10.7196/samj.2019.v109i2.13344	
Please pay attention in the English language used. The English language must be checked by professional language editor. Few examples:<br>Line 52-54, the language should be revised, also the sentence in incomplete. “ We need to explore environmental factors influencing health outcomes, inform the context-based interventions	Corrected grammar mistakes throughout the manuscript<br><br>There is a need to explore environmental factors that influence health outcomes to inform context-based interventions for people with disabilities in South Africa.	Line 51-52

for people with disabilities in South Africa, there.<br>In the methods part line 104 “We used a qualitative method was used to explore the environmental factors influencing the prevention of SHCs in people with SCI”.	We used a qualitative design to explore the environmental factors influencing the prevention of SHCs in people with SCI.	Line 103
**Reviewer**		
Line 119 - OPD – outpatient department? Please define	Changed to outpatient medical clinic	Line 119

Thank you for considering these corrections.

Mrs. Sonti Pilusa ___________
