# Supplementary material for: Environmental factors influencing the prevention of secondary health conditions among people with spinal cord injury, South Africa
Source: PLoS One. 2021 Jun 25;16(6):e0252280. doi: 10.1371/journal.pone.0252280 (PMC8232458; doi:10.1371/journal.pone.0252280)
Supplement: Attachment — Submitted filename: Response to Reviewers.docx [file pone.0252280.s001.docx]

**Table 1: Editors comments**

**Corrections -** highlighted in grey

| **Editors Comments** | **corrections** | **Section** |
| --- | --- | --- |
| 1. Please ensure that your manuscript meets PLOS ONE's style requirements, including those for file naming. | Revised |  |
| Please include additional information regarding the interview guide used in the study and ensure that you have provided sufficient details that others could replicate the analyses.  For instance, if you developed a guide as part of this study and it is not under a copyright more restrictive than CC-BY, please include a copy, in both the original language and English, as Supporting Information.  In addition, please include any further details about the development and validation of this tool. | Details on the interview guide included in the data collection section  Supplementary document added  Details on the interview guide included in the data collection section | Line 126-131 |
| We note that you have indicated that data from this study are available upon request. PLOS only allows data to be available upon request if there are legal or ethical restrictions on sharing data publicly. For information on unacceptable data access restrictions, please see <http://journals.plos.org/plosone/s/data-availability#loc-unacceptable-data-access-restrictions>.  If there are no restrictions, please upload the minimal anonymized data set necessary to replicate your study findings as either Supporting Information files or to a stable, public repository and provide us with the relevant URLs, DOIs, or accession numbers. Please see <http://www.bmj.com/content/340/bmj.c181.long> for guidelines on how to de-identify and prepare clinical data for publication. For a list of acceptable repositories, please see <http://journals.plos.org/plosone/s/data-availability#loc-recommended-repositories> | Added file as supporting information |  |
| Please amend the manuscript submission data (via Edit Submission) to include author Hellen Myezwa. | Amended |  |
| Please amend your list of authors on the manuscript to ensure that each author is linked to an affiliation. Authors’ affiliations should reflect the institution where the work was done (if authors moved subsequently, you can also list the new affiliation stating “current affiliation:….” as necessary). | Corrected |  |
| Please include a separate caption for each figure in your manuscript. | Figure removed |  |
| Please kindly address the reviewer's comments and also proof read the word to correct any English errors. | Revised |  |

**Table 2: Reviewers comments**

| **Reviewers comments** | **Corrections** | **Section** |
| --- | --- | --- |
| **General comments** | | |
| When referring to your participants with SCI, please refer to them as “participants”, rather than “patients.” While it is appropriate to use the term “patient” when individuals are in-hospital, or receiving direct medical or rehabilitative care, people with SCI who have enrolled in your study and who are presumably individuals in the community should be referred to as participants to reflect participatory nature of their study participation. Please refer to: Harvey, Lisa A. 2019. “Words Matter. Spinal Cord Asks Authors to Choose Their Words Carefully.” Spinal Cord 57(4):257–257. | Corrected throughout the manuscript  Patients with SCI replaced with participants or people with SCI |  |
| Your manuscript states that studies of environmental impacts on SHC prevention are limited in low-middle income countries. There is quite a lot in the literature for higher income countries, and persistent barriers arise regardless of the type of health care system, but for different reasons. That said, what are the underlying causes of your findings that are specific to how rehabilitation / health care is delivered in South Africa? As this is an international journal, your readership may not have a base understanding of your system. Reading your manuscript, I wanted to have a better understanding of the differences between your healthcare system and the one in my country. Please include a concise but informative description of how it works in your country (Private system? Public system? Combination of both? Who typically pays for what and when?). I encourage you to think broadly about the entire welfare regime – in other words – how your nation protects its most vulnerable not only in terms of health care, but also social resources for community living. Then, in the discussion, you can speculate how your results are tied to the structural barriers imposed by the health and welfare system, as appropriate. | Revised the introduction and added a section on the South African context and the health care system | Line 67-101 |
| • You introduce new results / material in the discussion and the conclusion (a figure). Please restructure appropriately. | Removed the figure |  |
| **Introduction** | | |
| • Lines 64-65, surprising that respiratory complications are missing from list of secondary health conditions, unless that is less of a problem in South Africa than in other regions. | Revised | Line 60-65 |
| You discuss the Guilcher study, and then allude to differences between Canadian and South African health care systems. As I indicated under general comments, a better understanding of the South African system would strengthen your manuscript. While you indicate that South Africa does not have national health insurance, you indicate that 84% are reliant on the public sector, presumably supported by the government. Please clarify how it generally works, including information about the other 16% - are they wealthy and pay privately? • There are persistent links between disability and poverty world-wide, in low income to high income countries. You indicate that a large proportion of the general population in South Africa lives in poverty. Can you comment on the proportion of people with disabilities living in poverty in South Africa? | Revised the introduction and added a section on the South African context and the health care system | Line 67-101 |
| **Methods** | | |
| Please provide more detail about recruitment, were prospective participants personally invited (if so, by whom), were there flyers, did you go to the therapy gym to recruit therapist participants, did SCI participants identify the caregiver participants, etc. | Added a section on recruitment | Line 126-129 |
| Describe purposive recruitment relative to your recruitment goals. Did you seek to recruit a wide range of injury levels, etc.? What other characteristics were considered? | Added in | Line 116-122 |
| Is it possible to include your interview guide as an appendix, or provide examples of questions / topics that were addressed? | Included topics covered and added the interview guide as a supplementary document | Line 126-129 |
| Line 109: Did all interviews last 60 minutes? In results, may want to indicate average length and range. | Revised the sentence | Line 134 |
| Did you begin coding with a preliminary set of codes from your interview guide? You discussed the ICF in the introduction. Did you use the ICF as a theoretical framework and if so, how did it influence your analysis? | Revised | 140-144 |
| **Results** | | |
| You have a very low number of participants with cervical level SCI. Please provide (in the discussion as relevant) reasoning for this – I wonder if people with higher level injury experience greater barriers returning to the hospital for follow-up and were thus less likely to be recruited. | Added a possible reason for low number of participants with cervical level SCI in the paragraph on the study limitations | Line 339-348 |
| Line 167: Please de-identify the name of the hospital / rehab center. | Revised –removed the name in the quote | Line 191 |
| Your section on Health care system inefficiencies would benefit, as previously suggested, from a brief but informative overview of how health care and rehabilitation are managed in South Africa. | Overview of the health system outlined in the introduction |  |
| The latter half of the results section gets a bit thin, with several headings without substantive content or thick description. | Revised | Line 203-250 |
| **Discussion** | | |
| In general, I think your results are important, but not surprising to people who work in SCI. Most if not all of the issues you report are experienced by people with SCI, to some extent, even in wealthier countries, but for different reasons. Since you acknowledge that there is a dearth of information in low-middle income countries about environmental factors that influence SHC prevention, you could spend some time in the discussion addressing why the results are the same or different (more below). | Revised the whole discussion section |  |
| Your discussion section should lead off with your most important finding(s), then include additional literature as relevant. As currently written, you begin reviewing literature without context to your findings. Indicate how your findings are congruent or incongruent with the studies you reference in the first paragraph | Revised | Line 256-258 |
| Lines 261-263 – this is a result, and should be reported under results in order to be addressed in the discussion | Revised | Line 263-265 |
| It is great that you discuss CRPD and SDGs. Since South Africa has ratified the CRPD, issues experienced by people with SCI can be addressed through UN reporting processes – please discuss how this has the potential to improve rights of people with disabilities. | Added a section on how the issues experienced by people with SCI can be addressed | Line 329-337 |
| Since the goal of your manuscript is to investigate SHC prevention in a lower-income region, it seems important for you to address / speculate about the root causes of your findings. | Added the reasons for the findings in the discussion section |  |
| What contributes to the shortage of resources (meds, supplies, assistive technology, etc.)? Lack of government investment? Again, it will be helpful for the reader to have a basic understanding of the South African health care system. For example, in the United States, access barriers can arise from insurers’ unwillingness to pay for certain resources, and if a person cannot pay privately, they do without. Are the shortages in South Africa different in that resources are unavailable even if someone was wealthy enough to pay privately? | Added a section on the South African health care in the introduction |  |
| Similarly, regarding prevention and care – what contributes to your reported non-compliance of health workers to various protocols – are they onerous to follow, lack of knowledge about their existence? What are the institutional influences that contribute? | Added the reasons Discussed the reasons | 302-305 |
| In general, you could shorten the discussion somewhat by concisely addressing the issues specific to your findings and your overall goal of examining environmental barriers to SHC prevention in a lower-income nation. | Revised |  |
| Conclusion • Conclusion: You introduce new material in the conclusion – Figure 1. This material would be better placed in the discussion, or perhaps even results section | Removed the figure |  |
| **References** | | |
| • Please correct reference #9 | Revised |  |
